# The Fort Collins Commuter Study: Impact of route type and transport mode on personal exposure to multiple air pollutants

**DOI:** 10.1038/jes.2015.68

**Published:** 2015-10-28

**Authors:** Nicholas Good, Anna Mölter, Charis Ackerson, Annette Bachand, Taylor Carpenter, Maggie L Clark, Kristen M Fedak, Ashleigh Kayne, Kirsten Koehler, Brianna Moore, Christian L'Orange, Casey Quinn, Viney Ugave, Amy L Stuart, Jennifer L Peel, John Volckens

**Affiliations:** 1 Department of Mechanical Engineering, Colorado State University, Fort Collins, Colorado, USA; 2 Department of Environmental and Radiological Health Sciences, Colorado State University, Fort Collins, Colorado, USA; 3 Department of Environmental Health Sciences, Johns Hopkins University, Baltimore, MD, USA; 4 Departments of Environmental and Occupational Health, and Civil and Environmental Engineering, University of South Florida, Tampa, Florida, USA

**Keywords:** air pollution, carbon monoxide, commute, particle number, particulate matter, traffic

## Abstract

Traffic-related air pollution is associated with increased mortality and morbidity, yet few studies have examined strategies to reduce individual exposure while commuting. The present study aimed to quantify how choice of mode and route type affects personal exposure to air pollutants during commuting. We analyzed within-person difference in exposures to multiple air pollutants (black carbon (BC), carbon monoxide (CO), ultrafine particle number concentration (PNC), and fine particulate matter (PM_2.5_)) during commutes between the home and workplace for 45 participants. Participants completed 8 days of commuting by car and bicycle on direct and alternative (reduced traffic) routes. Mean within-person exposures to BC, PM_2.5_, and PNC were higher when commuting by cycling than when driving, but mean CO exposure was lower when cycling. Exposures to CO and BC were reduced when commuting along alternative routes. When cumulative exposure was considered, the benefits from cycling were attenuated, in the case of CO, or exacerbated, in the case of particulate exposures, owing to the increased duration of the commute. Although choice of route can reduce mean exposure, the effect of route length and duration often offsets these reductions when cumulative exposure is considered. Furthermore, increased ventilation rate when cycling may result in a more harmful dose than inhalation at a lower ventilation rate.

## INTRODUCTION

Air pollution is a leading cause of disease and premature death in many countries.^1^ Despite recent reduction in air pollution levels in the developed countries,^2, 3^ evidence suggests that no safe threshold of exposure exists.^4^ Transport is a major source of air pollution,^5^ and living in proximity to major roads has been associated with increased risk of exposure and adverse health.^5^ Commuters appear to be at particular risk because of their daily exposure to traffic-related air pollution.^5^ Data from the 2009 American Community Survey suggests that a typical commuter in the United States would spend 1.2 years of their working lifetime commuting.^6^

Commuting by car is one of the most popular transport modes in the United States and Europe and is increasing elsewhere.^5^ Commuting by bicycle is an option available to many people and is increasingly encouraged as a healthy and low-emission alternative to driving.^7^ However, studies have suggested that cyclists may experience increased air pollution exposure and, because of their higher minute ventilation, substantially higher intake compared with drivers (see, e.g., Hatzopoulou *et al.*^8^). Evidence on air pollution exposures and related health effects specifically for cyclists, however, is limited, and introduces uncertainty into calculations for estimating the net health cost–benefit of switching from driving to cycling.^9^

The major strategies to reduce the adverse health effects of air pollution have evolved around minimizing emissions. An alternative (non-emissions reductions driven) approach is to reduce exposure by providing and facilitating behavioral choices that will result in lower exposures. By understanding the relationship between the choices commuters make and their exposure, it may be possible to reduce exposure by informing behavior and adapting urban infrastructure. A number of studies^10, 11, 12, 13, 14^ have investigated pollution levels on different routes, suggesting that routes can be chosen to reduce exposure. However, studies of actual commuters making realistic choices regarding route and mode are limited, and no studies have incorporated both cycling and driving in a non-prescribed (uncontrolled) setting. This study employed a crossover design on a panel of commuters living and working within Fort Collins, Colorado, United States, to assess the impact of switching transport mode from car to bicycle and of switching from direct routes to alternative (lower trafficked) routes on exposure to traffic-related air pollutants. The city of Fort Collins offered an ideal study domain to achieve these objectives; over 500 km of on-road cycling lanes and multi-use paths^15^ exist within the 78 km^2^ city limits.

## METHODS

### Study Population

The study consisted of volunteers who lived in the Fort Collins area and who commuted a minimum distance of 1.5 miles (2.4 km) from their home to their workplace. Inclusion criteria were the following: 18 to 65 years old, valid driver's license, current non-smoker, and no regular exposure to occupational dust and fumes. All participants provided written informed consent, and the Colorado State University Institutional Review Board approved all study procedures. Subjects received compensation for participation and access to their identifiable data is restricted.

### Study Location

Fort Collins, Colorado, United States, is a mid-size city located 100 km north of Denver, Colorado. Fort Collins has a population of 155,400 and a population density of 1060 km^−2^.^16^ Background levels of air pollutants are generally below US regulatory standards within the city, with motor vehicles representing a major source.^17^ Fort Collins has an extensive on-road cycling network that facilitates cycling across the entire city, as well as several long off-road cycle paths. The city was awarded platinum Bicycle Friendly Community status by the cycling advocacy group *The League of American Bicyclists* in 2013 in recognition of its efforts to promote safe cycling.^18^

### Commutes

Data collection took place from September 2012 to February 2014. Each participant was asked to complete 8 commute days within a 4–12-week period (to reduce seasonal effects on exposure): four cycling and four driving (both morning and evening each day) using their own vehicles. In consultation with study staff, each participant identified a direct and alternative route to follow. The direct routes generally followed arterial and larger collector (higher traffic) roads; the alternative route generally followed collector roads and paved, off-road cycle paths where available. Routes were chosen in conjunction with each participant. Participants were presented with direct and alternative route options. For cycling, the highest traffic count option was not chosen if the participant would never consider cycling on the busiest roads. Practical alternative routes were chosen such that the commute duration was not excessively long (within 0.3 miles on average) compared with direct routes. Each route and mode was repeated two times in a random sequence. Participants were asked to start their morning commute between 0700 and 0900 h and their evening commute between 1630 and 1800 h. Commutes took place on Tuesdays and Thursdays. Aborted or failed commutes (e.g., because of adverse weather, wrong route, start time, or instrument failure) were repeated after an individual completed their remaining sequence of commutes.

### Exposure Assessment

Participants carried a backpack containing instruments that measured environmental, spatial, and physiological variables, including personal exposure to multiple pollutants. Particulate matter <2.5 *μ*m in aerodynamic diameter (PM_2.5_) was sampled via a size-selective inlet (PEM, SKC, Eighty Four, PA, USA) and split between a nephelometer (pdr-1200, Thermo Fisher Scientific, Waltham, MA, USA) sampling at 3.8 l/min (OMNI 400, Mesa Labs, Butler, NJ, USA) and an aethalometer (MicroAeth, AethLabs, San Francisco, CA, USA) sampling at 0.2 l/min. The inlet was mounted on the backpack's shoulder strap to sample air within the participant's breathing zone. Particles were collected on a Teflon filter (PallFlex, Pall, Port Washington, NY, USA) downstream of the nephelometer. For a subset of participants, particle number concentration (PNC) was measured via a size-selective (PM_1.0_) inlet at 1 l/min (Disc Mini, Matter Aerosol AG, Switzerland). The PNC instrument was randomly assigned to an initial participant for all their commutes and when available assigned to a new participant. Carbon monoxide (CO) was sampled passively via an electrochemical sensor mounted on the side of the backpack (T15n, Langan Products, San Francisco, CA, USA). Sensors mounted on the top of the backpack (MSR Electronics, GmbH, Switzerland) measured temperature, relative humidity, light intensity, and movement. The participant's movement and heart rate were measured via a chest-mounted monitor (Actiheart, CamNtech, UK). The participant's location was tracked by a global positioning system (GPS) receiver (BT1000XT, Qstarz International, Taiwan). Data were logged at 10 s resolution or higher for all instruments. Black carbon (BC) data were corrected for loading, CO data were corrected for temperature, and PM_2.5_ data were corrected for humidity artifacts and non-linear instrument response at low concentrations (see Supplementary Material, p. 3).

Backpacks were given to participants the day before they followed the prescribed commute and returned the day after; pollution data analyzed here included only the morning and evening commuting times. The study ran through all seasons, and commutes were not scheduled during holidays to avoid unusual traffic patterns. Participants were asked to wear the backpack when cycling and place it on the passenger seat when driving. Compliance with these instructions was assessed by matching data from the movement sensor in the backpack with data from the movement sensor worn by the participant, using a simple binary moving and non-moving metric. Non-compliant commutes that occurred outside the start time window, or when the backpack was not carried, or when route deviation occurred (determined via GPS) were excluded from the analysis (see Supplementary Material, p. 4). Participants were given a questionnaire to fill out each commute day that included a log of their vehicle type and usage for each commute (including windows, heating, air conditioning, and air circulation). The mean (time-weighted average) and cumulative exposure to each measured air pollutant was calculated for each commute.

### Statistical Analyses

All analyses were performed in SAS version 9.3 (SAS Institute, Cary, NC, USA). The study samples size was chosen *a priori* by examining minimum detectible *r*^2^ values and power for a linear regression. Power calculations indicated at least 80% power to detect expected contrasts in air pollution exposure for 45 study participants completing a total of 720 commutes. Study power was lower for PNC measurements (219 total commutes). We calculated descriptive statistics for participant demographics and commuting routes. Morning (AM) and evening (PM) commutes were evaluated separately, and all pollutants were analyzed in separate models. Linear mixed models were used to analyze the effect of route and mode on the mean and cumulative exposure measured during the commute. To account for the repeated nature of the study design, a subject identifier was included as a random intercept. We evaluated mode (bicycle, car; car as the reference) regardless of route type; in a separate model we included mode, route, and an interaction term between route and mode to evaluate the following four categories of route/mode combinations: direct car, alternative car, alternative bicycle, and direct bicycle (direct car as the reference). The untransformed data did not me*et al*l of the model assumptions; therefore the following steps were used to transform the dependent variables: (1) one unit was added to all values; and (2) the resulting values were natural log transformed.^19^ All results are presented as mean percent differences with 95% confidence intervals (CIs) compared with the reference.^20^

Additional analyses included ambient temperature, ambient relative humidity, and ambient pollution (ambient CO in the personal CO model; ambient PM_2.5_ for all other pollutants) from Fort Collins' regulatory monitoring sites (EPA sites 840080691004 and 840080690009) in the model. The results did not change meaningfully with these variables included and therefore they were not included as covariates in the final model. We also stratified by season, ambient temperature, ambient relative humidity, and ambient pollution.

## RESULTS

**Figure 1:**
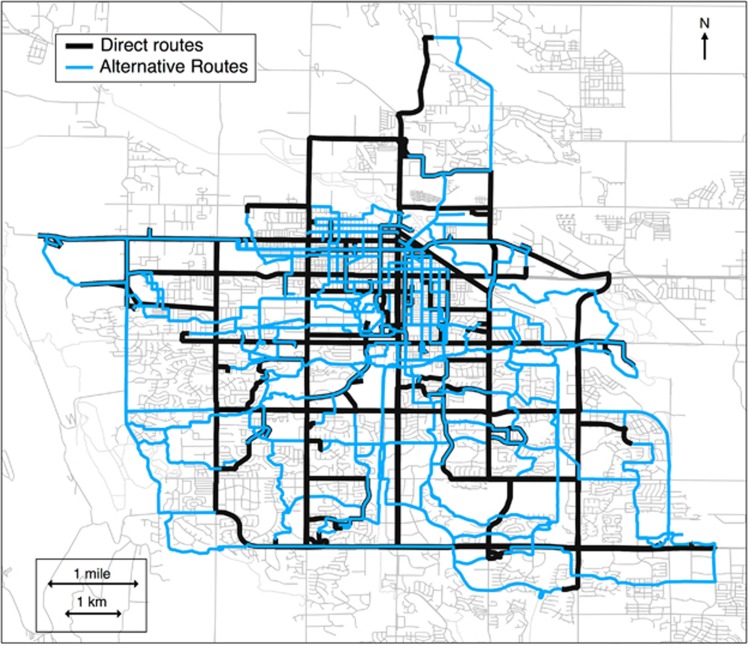
Map of Fort Collins showing the direct (black) and alternative (blue) routes taken by the study participants.

A total of 45 participants were recruited into the study. Of these, 41 participants completed the 8-day sequence, and 4 participants partially completed it. Of the 41 participants, 22 participants completed supplementary commute days because of instrument or compliance failure. In total, 381 days of data were collected, including 678 valid commutes (350 morning and 328 evening), covering routes across the entire city as illustrated in Figure 1. In all, 30 planned morning and 52 planned evening commutes were aborted or invalid because of issues such as weather, subject compliance, and instrument failure (see Supplementary Material, p. 4). After quality assurance (removal of data because of non-compliance and instrument failure), the number of morning/evening commute pollutant measurements was 315/292 for BC, 314/294 for CO, 299/276 for PM_2.5_, and 110/109 for PNC. Of the participants, 53% (*n*=24) were female, and the mean age was 38.8 years (SD=12.4 years; range 22–61 years). Mean (SD) commute length was 6.3 (3.4) km for the car direct routes (range, 1.4–17.8 km), 6.6 (3.7) km for the car alternative routes (range, 1.6–17.6 km), 6.4 (3.4) km for the direct bicycle routes (range, 1.4–17.5 km), and 6.5 (3.5) km for the alternative bicycle routes (range, 1.6–17.7 km). The mean (SD) duration of the commutes was 16 (7) min, 17 (8) min, 23 (11) min, and 25 (11) min, respectively. All participants drove modern (1990–2013) gasoline-powered vehicles, four of which were gas-electric hybrids. Consistent patterns in exposure were generally observed in the morning and evening commutes. Exposure gradients, for example the difference in mean BC, CO, and PM_2.5_ exposure when driving and cycling, tended to be slightly smaller (but with overlapping CIs) in the evening than the morning (Table 1 and Supplementary Table S1). Results for evening commutes are shown in the Supplementary Material, Supplementary Tables S1–S3, and Supplementary Figure S3.

### Cycling *vs* Driving (All Routes Combined)

**Figure 2:**
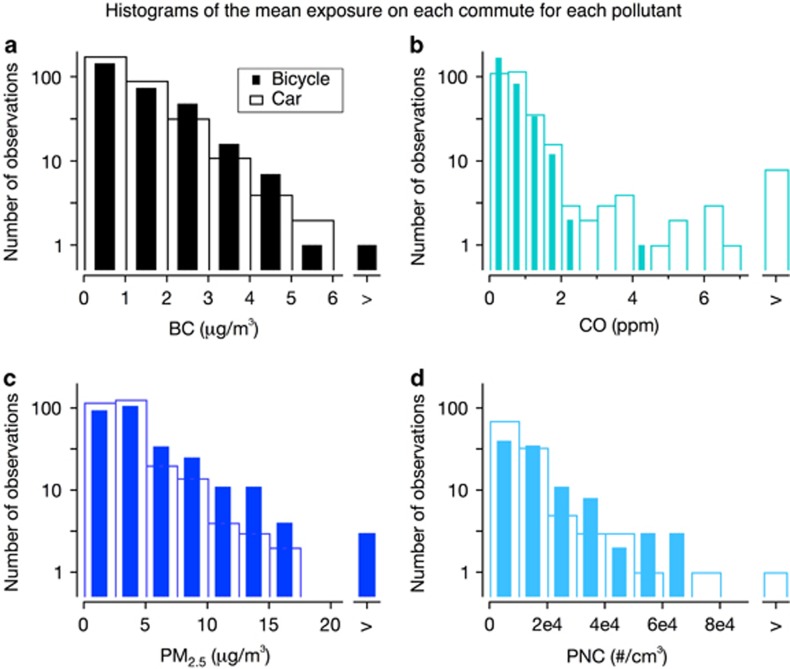
Histograms of participants. Mean BC (**a**), CO (**b**), PM_2.5_ (**c**), and PNC (**d**) exposure (morning and evening commutes combined).

Distributions of personal exposure as a function of mode (cycling and driving) and pollutant are presented in Figure 2. Cycling resulted in higher mean exposure levels to particulate pollution than driving during both morning and evening commutes (Table 1 and Supplementary Material and Supplementary Table S1). For example, cycling resulted in +13% (95% CI: +3, +24) higher mean BC, +25% (95% CI: +12, +39) higher mean PM_2.5_, and 41% (95% CI: +3, +94) higher mean PNC exposures during morning commutes. When considering cumulative exposure, the longer durations of cycling commutes tended to magnify these differences. For example, cycling resulted in +92% (95% CI: +56, +136) higher cumulative BC, +96% (95% CI: +68, +128) higher cumulative PM_2.5_, and +123% (95% CI: +58, +216) higher cumulative PNC exposure during morning commutes as compared with driving. Unlike the particulate pollutants, mean CO exposure was lower when cycling: −19% (95% CI: −25, −12) during morning and −16% (95% CI: −22, −9) during evening commutes (Table 1 and Supplementary Material and Supplementary Table S1). For cumulative CO exposure cycling exposure was no longer lower than driving exposures (i.e., the difference between cycling and driving was attenuated when considering cumulative exposures).

### Alternative *vs* Direct Routes (Within Mode)

When cycling, exposures to BC and CO were lower when on alternate routes compared with direct routes for both morning and evening commutes and for both mean and cumulative metrics (Table 2 and Supplementary Material and Supplementary Table S2). For example, across the morning commutes we observed −23% (95% CI: −13, −32) lower mean and −35% (95% CI: −51, −12) lower cumulative BC levels for the alternative bicycle routes compared with the direct bicycle routes. The results for driving were similar. Driving an alternate route produced lower mean exposures to BC and CO compared with driving a direct route. There was little difference observed for PNC and PM_2.5_ when comparing direct and alternative routes. Driving with windows opened or closed did not appreciably change these results (data not shown). This result suggests that a vehicle offers some protection from particle pollution compared with cycling. However, factors such as the cabin air settings, whether the window(s) were partially or fully open, seasonal differences, and the within-person analysis make it difficult to probe the question in more detail here.

### Differences between Route/Mode Combinations

**Figure 3:**
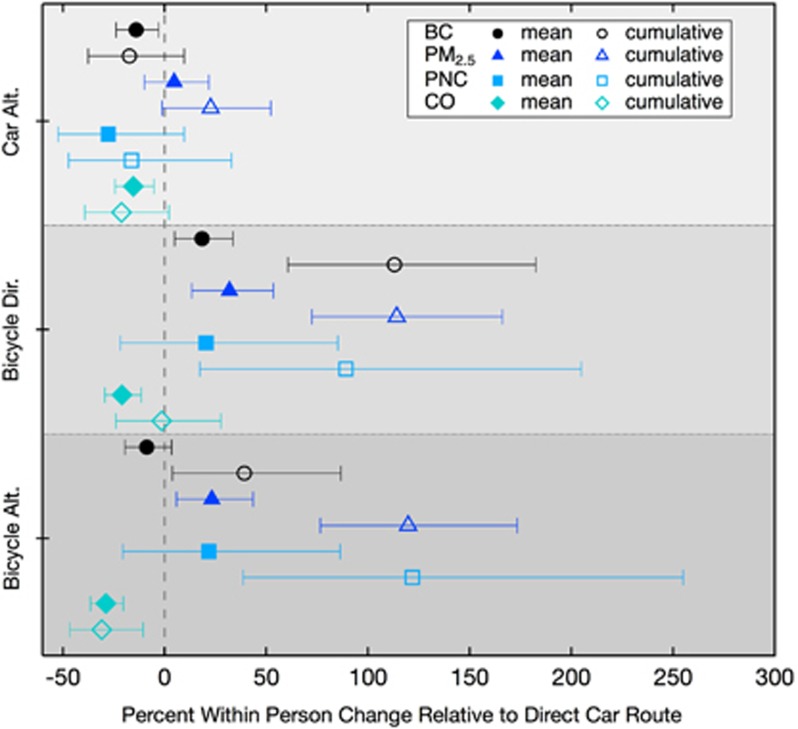
Mean percent within-person difference in mean and cumulative BC, PM_2.5_, and PNC exposures when driving an alternative route (car alt.), cycling a direct route (bicycle dir.), and cycling an alternative route (bicycle alt.) compared with driving a direct route.

The estimated differences in personal exposure for direct cycling routes and alternative cycling routes, compared with the direct car route, are presented in Figure 3 and Table 3. Commuters tended to experience higher mean particle exposures (BC, PM_2.5_, PNC) when cycling on direct routes, whereas mean CO exposures were highest when driving along direct routes. For example, mean BC exposure was +18% (95% CI: +5, +34) higher, and cumulative BC exposure was +113% (95% CI: +61, +182) higher, when comparing cycling with driving along direct routes in the morning. Patterns were not consistent when comparing alternate cycling with direct driving routes. Mean exposures to CO were lower for cycling an alternative route (*vs* direct driving) for both morning and evening commutes. Cumulative BC exposures were higher when cycling on alternate routes (*vs* direct driving), whereas cumulative CO exposures were still lower when cycling alternative routes compared with direct driving. Mean and cumulative exposure to PNC and PM_2.5_ tended to be higher for cycling on an alternate route compared with driving a direct route (morning and evening).

Results from models stratifying by ambient temperature, ambient relative humidity, and ambient pollution were not meaningfully different than results from our primary models. In models stratified by season, results for Spring, Summer, and Fall were similar to results from primary models. For mean and cumulative BC exposure, the differences between cycling direct and driving direct were even larger in the winter than in the primary models; similar results were observed for cycling alternative compared with driving direct. For mean and cumulative CO exposure, the lower values observed for cycling direct compared with driving direct in the primary models were attenuated in the winter; similar results were observed for cycling alternative compared with driving direct.

## DISCUSSION

The choices of commuting route and mode can have important consequences on a person's lifetime exposure to traffic-related air pollution. With an estimated 1.2 years of commuting exposure across a working lifetime, such choices have the potential to reduce the risks of chronic diseases associated with air pollution exposure. In the present study cyclists tended to experience higher exposures to the various forms of particulate air pollution (BC, PM_2.5_, and PNC) than drivers but lower exposure to CO. Key to this discussion, however, is the difference in duration of cycling *vs* driving commutes because cumulative exposure is a function of both exposure intensity and commute duration. Longer commute times, regardless of route type, tend to increase cumulative exposures; this difference was especially evident for cycling. Even though cyclists' mean particulate exposures were reduced on alternative routes, the longer duration of these routes increased cyclist's cumulative exposures relative to driving. Alternative cycling routes were less effective at reducing mean PM_2.5_ exposure, likely because PM_2.5_ consists of primary and secondary pollutants from both regional and local sources.^21^ We observed the lowest particulate exposures for drivers who commuted along alternative routes; the lowest CO exposures were observed for cyclists on alternative routes.

Our results do suggest, however, that cyclists can reduce their overall exposure to BC by taking alternative routes along lower trafficked roads (*vs* cycling along higher trafficked roadways). The observed reductions in cyclist's mean BC exposures on alternative routes (~20% lower than direct routes) are similar to the 15% reduction in BC observed by Jarjour *et al.*^22^ and the 20–28% reduction in soot reported by Zuurbier *et al.*^23^ and Strak *et al.*^24^, respectively. The reduced CO exposure observed when cycling alternative routes (~10%) is similar to Jarjour *et al.*^22^ (12%). Small differences in mean PM_2.5_ exposure between direct/alternate cycling routes are also consistent with other studies.^22, 23^ The observed reductions in PNC exposure for evening commutes on alternative bicycle routes (−26% *vs* direct cycling) were similar to those observed in other studies (Cole-Hunter *et al.*^14^ (35%), Jarjour *et al.*^22^ (23%), Strak *et al.*^24^ (37%), and Zuurbier *et al.*^23^ (19%)); morning cycling commutes showed little difference in PNC exposure in our study. Other studies have shown that route characteristics (e.g., road type, traffic volume, the presence of a cycling lane, etc) explain some variability in exposure,^25, 26^ and it appears possible to design routes that reduce exposure.^11^ In this study, most of the alternative cycling routes were along on-road cycle lanes as opposed to multi-use cycle paths that are not contiguous with the roadway. When designing alternative routes, we attempted to make the route a realistic choice for the commuter (i.e., one that did not add substantial length compared with the direct route). As a result, the multi-use cycle paths were practical for only a small fraction of our participants; only 5 of our 45 participants had >50% of their alternative cycling route on a multi-use path (see Supplementary Material and Supplementary Figure S1).

Previous studies comparing particulate exposures between drivers and cyclists present mixed results.^27, 28, 29, 30, 31, 32^ Boogaard *et al.*^33^ and Panis *et al.*,^28^ for example, compared driving and cycling exposures to PNC and PM_2.5_ across multiple cities and found that the direction and magnitude of relative differences varied from city to city. Other studies, however, observed increased mean exposure to particulate pollutants when driving (e.g., BC (49%^34^), PM_2.5_ (12%^35^ and 17%^34^), PNC (6%^35^ and 36%^34^) and elemental carbon (41% summer and 44% winter^30^)) in drivers relative to cyclists on comparable routes. Local traffic patterns, fleet composition, vehicle operation, topography, and proximity to source (i.e., the tailpipe) all likely contribute to the relative differences in exposure between cyclists and drivers. An increase in mean CO exposure when driving is consistent with several previous studies.^32, 35^ The observed increase in mean CO exposure when driving (15% to 20% greater than cycling) is similar to Kaur *et al.*^35^ (15%) but lower than de Nazelle *et al.*^32^ (75% geometric mean). Additional studies comparing cycling and driving exposures to gaseous air pollutants have found driver's exposure is elevated by 60% to 75%.^13, 29^

The Fort Collins Commuter Study is one of the largest measurement data sets collected on commuter air pollution exposure to date, comprising 678 valid commutes. The study design focused on acquiring representative exposures of commuters on actual commute routes, with as little constraint on participant's behavior as was practical (most studies^12, 23, 32, 36, 37^ published to date have used artificial routes). This is also the first large study to evaluate both mode (driving *vs* cycling) and route (alternative *vs* direct) choices that represent realistic options for individual commuters. Time-resolved data were collected for multiple pollutants, over a full working day, with participants recruited in all seasons. Thus, morning and evening commutes could be compared, a question that has not been addressed by the majority of commuter exposure studies. Consistent patterns in exposure were generally observed in the morning and evening commutes; however, the differences between the route/mode combinations are typically smaller in the evening (see Supplementary Material and Supplementary Tables S1–S3). Pollutant concentrations observed here were generally toward the lower end of those reported elsewhere, and were comparable to previous studies.^22, 27, 29^ However, despite the relatively low pollution levels, we observed differences in exposures between both commuting modes and route type.

The study design required participants to keep a 6 kg (13.2 lb) backpack with them for 36 h on 8 occasions; this burden resulted in the recruitment of highly motivated participants who were likely to be regular cyclists. Our results are likely generalizable to commuters in other mid-sized cities, given the agreement between our results and other studies. The participant's commutes provided good spatial coverage of the city (Figure 1); however, commuting times were restricted to rush-hour intervals. Thus, our results may not be generalizable to off-peak commute times. Each route was chosen in conjunction with the participant. Therefore, roads on which they would not consider traveling were avoided; often the busiest roads were avoided altogether when cycling. The route selection method had the advantage of providing realistic choices that a commuter could make but did not necessarily maximize the exposure gradient between routes. Therefore, the effects of route and mode on exposure may not be as large as in studies where routes are preselected to provide a large contrast in exposure. The choice of route type based on road type and expected traffic counts could be optimized with better knowledge of on-road pollution levels and other contributing factors; however, the City's street layout was a limiting factor. Fort Collins' gridded street layout meant direct routes were fairly straightforward to plan, and for most participants comprised over 70% arterial roads (see Supplementary Material). Planning realistic alternative routes that avoided arterial roads was more difficult; 11 alternative driving and 6 alternative cycling routes comprised ⩾50% arterial roads by distance.

The study took place in a mid-sized US city where ambient pollution levels are generally low compared with larger cities where many previous studies on commuter air pollution have taken place. The study location therefore provides insight into exposures in an understudied region of the United States; however, not all of our observations may scale to larger cities. Exposures to multiple pollutants were measured; however, traffic-related emissions contain numerous other gases and specific particle types harmful to human health. The pollutants measured here are not unique to traffic sources, but on-road vehicles are expected to be the dominant source of BC and CO in Fort Collins.^17^

### Implications for Public Health

Given the ubiquitous and insidious nature of air pollution exposure, strategies are needed to help individuals reduce their daily intake. Such strategies can be impactful, because there is no “safe” level of air pollution exposure and because the largest marginal benefits to human health are hypothesized to occur when relatively low exposure levels are reduced further (given the log-linear nature of exposure–response for many health outcomes^38^). Replacing short car journeys with cycling has the potential to produce health benefits for both the individual (through increased exercise) and the general public (through reduced emissions).^39^ The benefits of switching to cycling may be particularly large in a country like the United States where car transit dominates and physical inactivity represents a major risk factor for disease.^40^ The present study, however, suggests that cyclists have higher air pollutant exposures than drivers, especially for particulate pollutants. The health risk to cyclists may be exacerbated by higher breathing rates, and this probably results in at least a doubling of their estimated inhaled exposure relative to driving.^41^ A population-level shift from driving to cycling will lower pollution emissions and also increase physical activity (see, e.g., Johan de Hartog *et al.*^9^). The benefit of increased exercise is likely to outweigh the risks because of increased air pollution intake caused by higher minute ventilation (and other risk factors) when cycling,^42^ especially in underactive members of the population. The relationship between minute ventilation rate and the harmful dose of pollution is an important uncertainty that when better understood could alter the health cost–benefit balance of cycling *vs* driving. The present study considered mean and cumulative exposure; analysis of the effects of minute ventilation and pollution inhalation is beyond the scope of this paper but will be considered in future manuscripts.

Routing cyclists away from the busiest roads is an obvious strategy for reducing exposure. Our results show that alternative cycling routes within the existing road infrastructure only result in limited exposure reduction. Furthermore, these mean exposure reductions are not great enough to overcome the effect of the longer commute duration (on cumulative exposure) and the protection from PM offered by air filtration in modern cars. This result stands out given that Fort Collins is touted as a cycling-friendly city. The majority of the alternative cycling routes were still mainly along roads with automotive traffic; only five commuters had a practical alternative cycling route comprising ⩾50% of off-road cycle paths (see Supplementary Material and Supplementary Figure S2). A larger reduction in exposure may have been achieved with greater separation of cyclists and drivers. The extensive cycling infrastructure in Fort Collins consists primarily of on-road cycle lanes; to reduce cyclists' exposure relative to driving, infrastructure that further separates cyclists from cars is likely needed. The gridded city layout common in the United States may pose particular difficulties in designing low-exposure routes, as intersections with heavier traffic on arterial roads are difficult to avoid when commuting. Urban infrastructure that moves cyclists further away from cars, such as off-road multi-use paths, is likely necessary to protect cyclists from undue exposure to traffic-related air pollution.

## Figures and Tables

**Table 1:** Effect of mode (cylcing vs. driving).

*Pollutant (metric)*	*Morning commute*		
	*% (95% CI)*	N	P*-value*
BC (mean)	+13 (+3, +24)	315	0.009
BC (cumulative)	+92 (+56, +136)	315	<0.0001
CO (mean)	−19 (−25, −12)	314	<0.0001
CO (cumulative)	−8 (−24, +11)	314	0.4
PM_2.5_ (mean)	+25 (+12, +39)	299	<0.0001
PM_2.5_ (cumulative)	+96 (+68, +128)	299	<0.0001
PNC (mean)	+41 (+3, +94)	110	0.03
PNC (cumulative)	+123 (+58, +216)	110	<0.0001

Abbreviations: BC, black carbon; CI, confidence interval; CO, carbon monoxide; PM_2.5_, particulate matter <2.5 *μ*m in diameter; PNC, particle number concentration.

Within-person difference in personal exposure between driving and cycling routes using driving routes as the reference (positive values mean cycling is higher).

**Table 2:** Effect of route type within mode.

*Pollutant (metric)*	*Morning commute*					
	*Bicycle (alt. vs dir.)*			*Car (alt. vs dir.)*		
	*% (95% CI)*	N	P*-value*	*% (95% CI)*	N	P*-value*
BC (mean)	−23 (−32, −13)	147	<0.0001	−14 (−24, −3)	159	0.02
BC (cumulative)	−35 (−51, −12)	147	0.005	−17 (−38, +10)	159	0.2
CO (mean)	−10 (−20, +1)	151	0.08	−15 (−24, −5)	157	0.004
CO (cumulative)	−30 (−46, −9)	151	0.009	−21 (−39, +2)	157	0.07
PM_2.5_ (mean)	−7 (−20, +9)	140	0.4	5 (−10, +22)	145	0.5
PM_2.5_ (cumulative)	+3 (−18, +28)	140	0.8	23 (−1, +52)	145	0.06
PNC (mean)	+1 (−35, +59)	39	1	−28 (−52, +10)	40	0.1
PNC (cumulative)	+17 (−29, +93)	39	0.5	−16 (−47, +33)	40	0.4

Abbreviations: alt., alternative route; BC, black carbon; CI, confidence interval; CO, carbon monoxide; dir., direct route; PM_2.5_, particulate matter <2.5 *μ*m in diameter; PNC, particle number concentration.

Within-person difference in personal exposure between alternative and direct routes within mode (car or bicycle) using the direct route as the reference (negative values mean the alternative route is lower than the direct route).

**Table 3:** Cycling compared with driving a direct route.

*Pollutant (metric)*	*Morning commute*					
	*Bicycle direct*			*Bicycle alternative*		
	*% (95% CI)*	N	P*-value*	*% (95% CI)*	N	P*-value*
BC (mean)	+18 (+5, +34)	160	0.006	−9 (−19, +3)	145	0.2
BC (cumulative)	+113 (+61, +182)	160	<0.0001	+39 (+4, +87)	145	0.03
CO (mean)	−21 (−29, −12)	158	<0.0001	−29 (−36, −20)	156	<0.0001
CO (cumulative)	−1 (−24, +28)	158	0.9	−31 (−47, −11)	156	0.005
PM_2.5_ (mean)	+32 (+13, +53)	144	0.0004	+23 (+6, +43)	141	0.007
PM_2.5_ (cumulative)	+114 (+72, +166)	144	<0.0001	+120 (+77, +173)	141	<0.0001
PNC (mean)	+20 (−22, +85)	39	0.4	+22 (−20, +86)	47	0.4
PNC (cumulative)	+89 (+17, +205)	39	0.01	+122 (+39, +255)	47	0.001

Abbreviations: BC, black carbon; CI, confidence interval; CO, carbon monoxide; PM_2.5_, particulate matter <2.5 *μ*m in diameter; PNC, particle number concentration.

Within-person difference in personal exposure comparing the bicycle commutes with the direct car route (positive values mean cycling is higher).
