# Supplementary material for: The Fort Collins Commuter Study: Impact of route type and transport mode on personal exposure to multiple air pollutants
Source: J Expo Sci Environ Epidemiol. 2015 Oct 28;26(4):397–404. doi: 10.1038/jes.2015.68 (PMC4848179; doi:10.1038/jes.2015.68)
Supplement: Supplementary Information [file jes201568x1.doc]

# Supplemental Material

The Fort Collins Commuter Study: Impact of route type and transport mode on personal exposure to multiple air pollutants

Nicholas Good^1,2^ PhD., Anna Mölter^2^ PhD., Charis Ackerson^2^ BS., Annette Bachand^2^ PhD., Taylor Carpenter^2^ BS, Maggie L. Clark^2^ PhD., Kristen M. Fedak^2^ MS., Ashleigh Kayne^2^ MS, Kirsten Koehler^4^ PhD., Brianna Moore^2^ MS, Christian L’Orange^1,2^ PhD., Casey Quinn^2^ MS., Viney Ugave^1^ MS, Amy L. Stuart^3^ PhD., Jennifer L. Peel^2^ PhD., and John Volckens^1,2^ PhD.

**Affiliations**:

^1^Department of Mechanical Engineering, Colorado State University, Fort Collins, CO, USA.

^2^Department of Environmental and Radiological Health Sciences, Colorado State University, Fort Collins, Colorado, USA.

^3^Departments of Environmental and Occupational Health, and Civil and Environmental Engineering, University of South Florida, Tampa, FL, USA

^4^Department of Environmental Health Sciences, Johns Hopkins University, Baltimore, MD, USA

**Correspondence**: Name: John Volckens, Address: 1374 Campus Delivery, Colorado State University, Fort Collins, Colorado, USA, 80523-1301 Telephone: 970-491-6341. Email: john.volckens@colostate.edu

# Table of contents

Supplemental Material [1](#__RefHeading___Toc303433214)

Table of contents [2](#__RefHeading___Toc303433215)

Data analysis [3](#__RefHeading___Toc303433216)

Participant compliance [4](#__RefHeading___Toc303433217)

Note on cancelled and rescheduled commutes [5](#__RefHeading___Toc303433218)

Route types [6](#__RefHeading___Toc303433219)

Evening Commute Data [8](#__RefHeading___Toc303433220)

References [11](#__RefHeading___Toc303433221)

# Data analysis

Black Carbon Data: Equivalent black carbon concentrations were derived from miniaturized aethalometer measurements (AE51, AethLabs, CA, USA). The aethalometers were operated at a nominal flow rate of 250 ml/min sub-sampling from a PM_2.5_ personal sampling inlet (PEM 761-203B, SKC Inc., Eighty Four, PA, United States). The instrument’s flow rate was measured (TriCal, Mesa Labs Inc., Butler, NJ, United States) prior to each deployment. A constant flow-rate correction factor was applied (initial set-point flow rate / measured flow rate) to the instrument’s internally measured flow rate. Data was logged at 0.1 Hz. The time series data was smoothed using a minimum delta attenuation of 0.05 (Hagler et al. 2011). A loading correction was then applied to the time series data to account for the effect of filter loading (Kirchstetter and Novakov 2007). An equivalent black carbon mass concentration was then calculated from the change in attenuation, assuming an attenuation cross section of 16.6 m^2^g^-1^.

Carbon Monoxide Data: Carbon monoxide mixing ratio was derived from a portable electrochemical device (T15n, Langan Products, San Francisco, CA, USA). The devices were calibrated periodically at 0 ppmV and 10 ppmV. The 0 ppmV calibration point was measured by sealing the inlet to the device for 5 minutes, allowing the CO in the system to react away. The 10 ppmV calibration point was measured by flowing 10 ppmV calibration gas (SKC Inc., Eighty Four, PA, United States) over the device for 5 minutes. The voltage to mixing ratio relationship was then derived using a linear fit. The cell response was adjusted for temperature using the on-board sensor (Langan Products Inc 2006)

Particle Number Data: Particle number was derived from a two stage diffusion classifier (DiscMini, Matter Aerosol GmbH, CH). Size distributions were reconstructed from the change in current across each stage by assuming two lognormal number size distributions with modal diameters of 30 nm for the first (diffusion) stage and 200 nm for the second (impaction) stage. Both lognormal distributions were assumed to have a standard deviation of 1.9.

Particle Mass Data: PM_2.5_ was measured using nephelometry (pDR-1200, Thermo Fisher Scientific Inc., MA, USA) and logged at 0.1 Hz. The instrument is known to underestimate at low concentrations therefore measured relative humidity adjusted (Chakrabarti et al. 2004) values (*Mass_meas_*) under 3 μg/m^3^ were adjusted (*Mass_adj_*) using the following formula derived from laboratory measurements of ammonium sulfate test aerosol compared to the mass derived from a scanning mobility particles sizer (3081, TSI Inc, USA):

$Mass_{adj}=Mass_{meas}+1.57\left(1.59/\left(1+\exp\left(\frac{1.98-Mass_{meas}}{0.80}\right)\right)\right)$ - Equation 1

# Participant compliance

Whether the monitoring backpack was carried on a commute was assessed by comparing accelerometer data measured by the Actiheart (worn by the participant on their torso) and the MSR145 (placed in the backpack) respectively (Figure S1, panels D and E) as a function of GPS location (Figure S1, panels A and B). When a commute is taking place it is obvious if the backpack is moving in concert with the participant by splitting the signals into two categories: moving and not moving, and matching them up. The Actiheart also contains a heart-rate monitor, which can confirm that the personal movement sensor is being worn. Comparison of the participants planned and actual was route determined by GPS (Figure S1, panels A and B). The GPS timestamp was used to ensure the commute started within the specified time window.


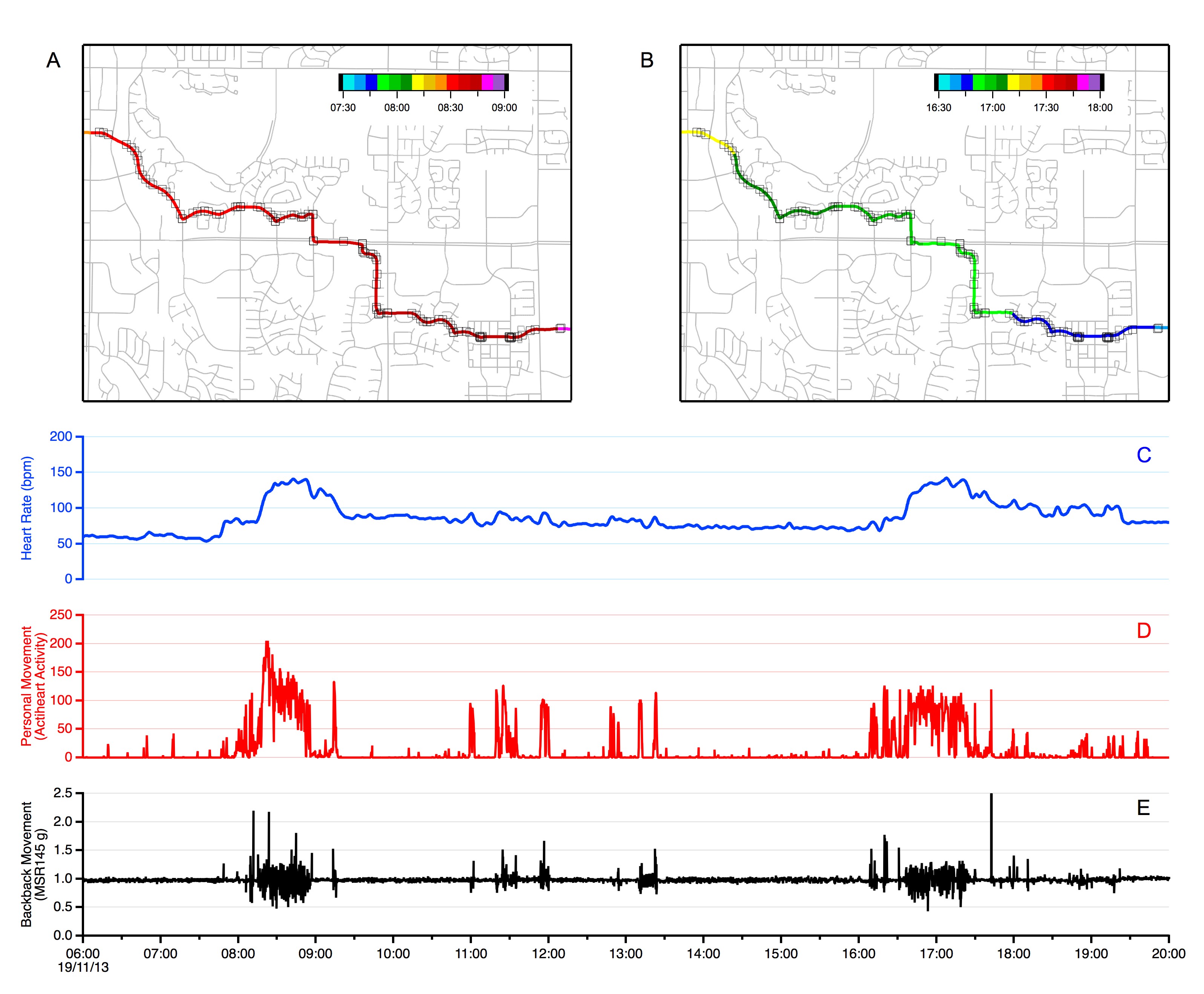


Figure S1 – Example of compliance assessment input data. Panels A and B show a planned and actual route section for a participant’s morning and evening commute colored by time. Panel C shows heart rate. Panel D shows the participant’s activity level. Panel E shows the backpack’s movement.

# Note on cancelled and rescheduled commutes

When commuting by bicycle was not possible due to adverse weather, all commutes on that day were cancelled to avoid biasing the results. Commutes cancelled for any reason were rescheduled to take place at the end of the participant’s random route-mode sequence.

# Route types

Supplemental Figure 2 illustrates the relative proportion of road types encountered during each participant’s planned commutes for all route and mode combinations, ordered by the fraction of arterial roads the commute comprised. Direct route choice was generally obvious owing to the Fort Collins’ gridded road layout. Direct car routes (Figure S2 A) were predominately along arterial roads running North-South and East-West. The fraction of collector or smaller roads taken on direct routes corresponds to the distance a participant’s home or workplace is from an arterial connection. Direct bicycle routes (Figure S2 C) were similar to their corresponding direct car routes, the main difference being a slightly lower fraction of major arterial roads. Cyclists often chose to avoid major arterial roads, especially those that lacked bicycle lanes; such routes were rarely considered, as they would not represent a realistic commute. Alternative car routes (Figure S2 B) were comprised of a larger fraction of smaller (non-arterial) roads than direct routes; however for some participants the fraction of arterial roads on alternative routes remains high (>50%). The reason some participants had a relatively high fraction of arterial roads on their alternative route was due to a lack of a reasonable (i.e. not excessively longer distance) lower trafficked alternative. A high fraction of arterial roads on alternative driving routes tended to occur on Lateral (East-West, West-East) commutes in some parts of the city. The alternative bicycle routes (Figure S2 D) were comprised of a modest fraction of off-road paths; however most routes were made up of a large fraction of sections on smaller roads expected to have low traffic counts. Despite a number of long sections of off-road cycle paths in the city, they often do not present a practical route choice.


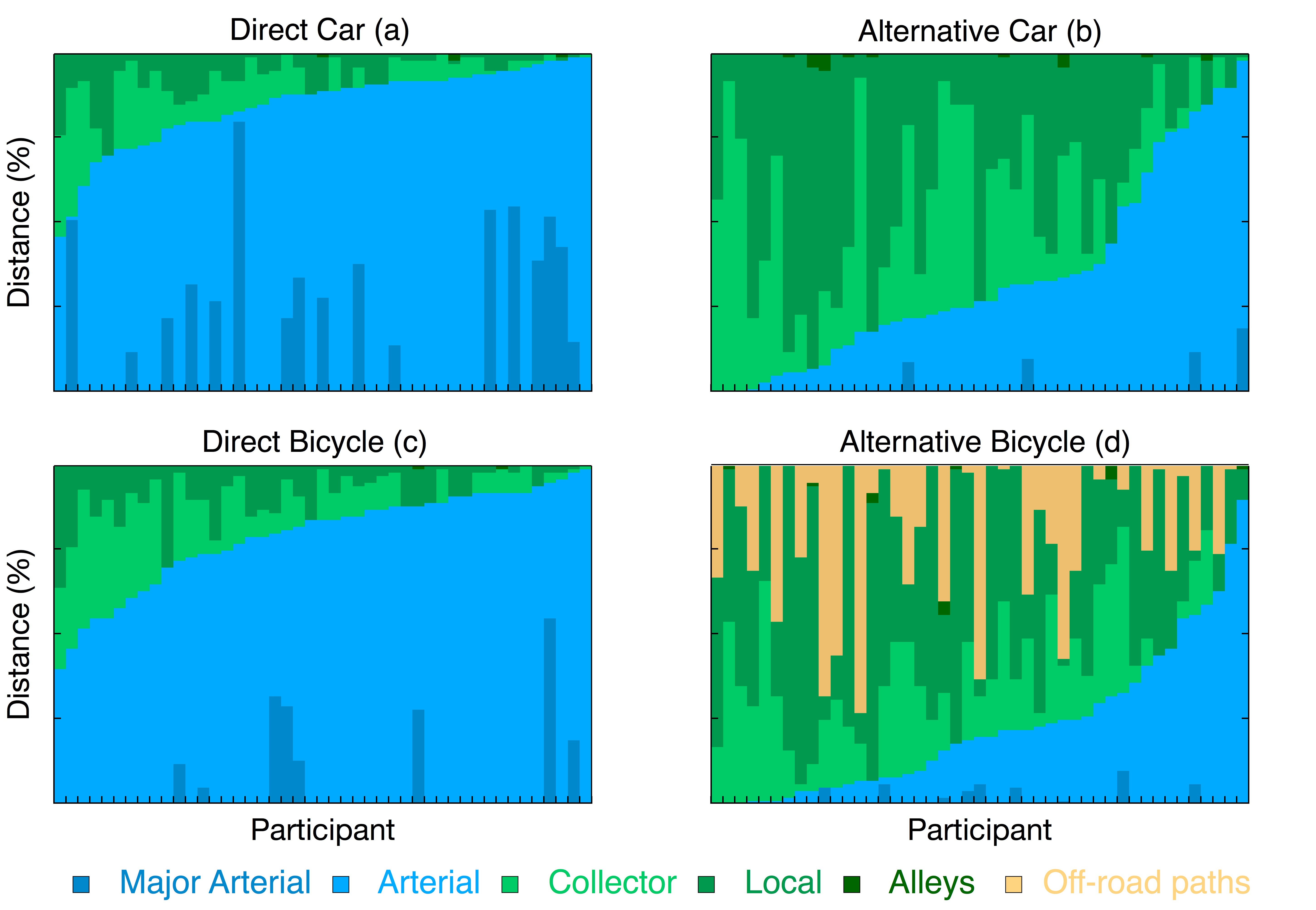


Figure S2 – The road type on each participant’s routes as the percentage of the route length. Panel (a) shows direct car routes, panel (b) shows alternative car routes, panel (c) shows direct bicycle routes and panel (d) shows the alternative bicycle routes.

# Evening Commute Data

Table S1 shows the comparison of all driving versus all cycling evening commutes. Table S2 shows the within-mode comparison of direct versus alternative evening commute routes. Table S3 shows the comparison of direct and alternative cycling routes to driving a direct route. Supplemental Figure S3 shows the evening commute within person change in mean and cumulative exposures for the different route-mode combinations relative to driving a direct route.


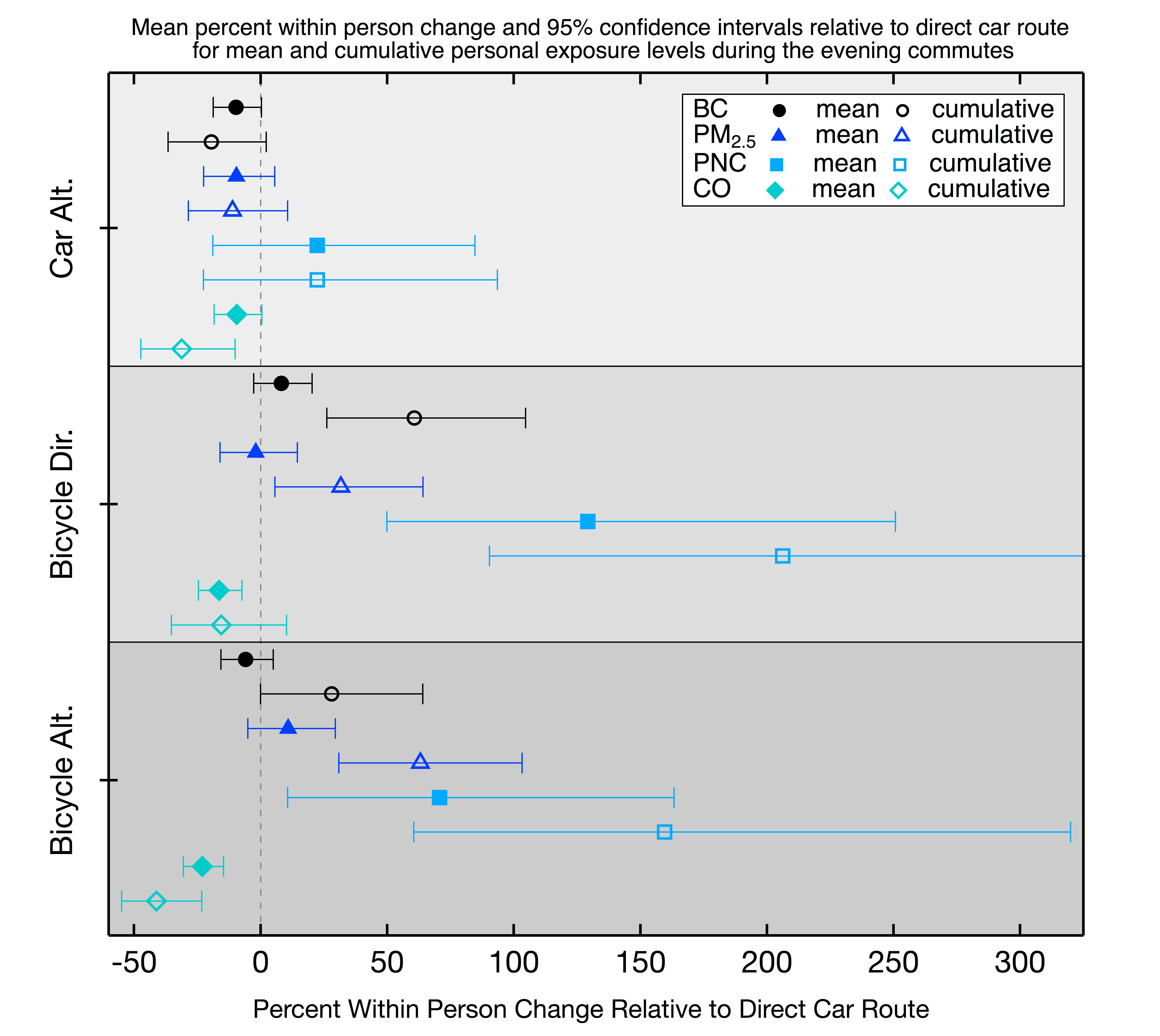


Supplemental Figure S3 - Change within person mean and cumulative BC, PM_2.5_ and PNC exposures when driving an alternative route (car alt.), cycling a direct route (bicycle dir.) and cycling an alternative route (bicycle alt.) compared to driving a direct route.

| **Supplemental Table S1. Effect of mode:** Within-person difference in personal exposure between driving and cycling routes using driving routes as the reference (positive values mean cycling is higher). | | | |
| --- | --- | --- | --- |
| Pollutant (Metric) | Evening Commute | | |
|  | % (95% CI) | n | p |
| BC (Mean) | +7 (-1, +15) | 292 | 0.1 |
| BC (Cumulative) | +61 (+35, +92) | 292 | <0.0001 |
| CO (Mean) | -16 (-22, -9) | 294 | <0.0001 |
| CO (Cumulative) | -15 (-30, +3) | 294 | 0.1 |
| PM_2.5_ (Mean) | +10 (-1, +23) | 276 | 0.09 |
| PM_2.5_ (Cumulative) | +56 (+34, +82) | 276 | <0.0001 |
| PNC (Mean) | +78 (+32, +140) | 109 | 0.0003 |
| PNC (Cumulative) | +153 (+81, +252) | 109 | <0.0001 |
| BC = Black Carbon, CO = Carbon Monoxide, PM_2.5_ = Particulate Matter less than 2.5 microns in diameter, PNC = Particle Number Concentration, CI = Confidence Interval. | | | |

| **Supplemental Table S2. Effect of route type within mode.** Within-person difference in personal exposure between alternative and direct routes within mode (car or bicycle) using the direct route as the reference (negative values mean the alternative route is lower). | | | | | | |
| --- | --- | --- | --- | --- | --- | --- |
| Pollutant (Metric) | Evening Commute | | | | | |
|  | Bicycle (alt vs. dir) | | | Car (alt vs. dir) | | |
|  | % (95% CI) | n | p | % (95% CI) | n | p |
| BC (Mean) | -13 (-22, -3) | 134 | 0.01 | -10 (-19, +0) | 139 | 0.06 |
| BC (Cumulative) | -20 (-38, +2) | 134 | 0.07 | -20 (-37, +2) | 139 | 0.07 |
| CO (Mean) | -8 (-17, +2) | 147 | 0.1 | -9 (-18, +0) | 142 | 0.06 |
| CO (Cumulative) | -30 (-47, -9) | 147 | 0.008 | -31 (-47, -10) | 142 | 0.006 |
| PM_2.5_ (Mean) | +13 (-3, +32) | 131 | 0.1 | -10 (-23, +6) | 126 | 0.2 |
| PM_2.5_ (Cumulative) | +24 (+0, +54) | 131 | 0.05 | -11 (-29,+11) | 126 | 0.3 |
| PNC (Mean) | -26 (-52, +14) | 42 | 0.2 | +22 (-19, +85) | 34 | 0.3 |
| PNC (Cumulative) | -15 (-47, +36) | 42 | 0.5 | +22 (-23, +93) | 34 | 0.4 |
| alt = Alternative Route, dir = direct route, BC = Black Carbon, CO = Carbon Monoxide, PM_2.5_ = Particulate Matter less than 2.5 microns in diameter, PNC = Particle Number Concentration, CI = Confidence Interval. | | | | | | |

| **Supplemental Table S3. Cycling compared to driving a direct route**. Within-person difference in personal exposure comparing the bicycle commutes to the direct car route (positive values mean cycling is higher). | | | | | | |
| --- | --- | --- | --- | --- | --- | --- |
| Pollutant (metric) | Evening Commute | | | | | |
|  | Bicycle Direct | | | Bicycle Alternative | | |
|  | % (95% CI) | n | p | % (95% CI) | n | p |
| BC (Mean) | +8 (-3, +20) | 138 | 0.1 | -6 (-16, +5) | 134 | 0.3 |
| BC (Cumulative) | +61 (+26, +105) | 138 | 0.0001 | +28 (+0, +64) | 134 | 0.05 |
| CO (Mean) | -16 (-25, -7) | 143 | 0.0007 | -23 (-31, -15) | 143 | <0.0001 |
| CO (Cumulative) | -16 (-35, +10) | 143 | 0.2 | -41 (-55, -23) | 143 | 0.0001 |
| PM_2.5_ (Mean) | -2 (-16, +15) | 124 | 0.8 | +11 (-5, +30) | 125 | 0.2 |
| PM_2.5_ (Cumulative) | +32 (+6, +64) | 124 | 0.01 | +63 (+31, +103) | 125 | <0.0001 |
| PNC (Mean) | +129 (+50, +251) | 31 | 0.0002 | +71 (+11, +163) | 36 | 0.02 |
| PNC (Cumulative) | +206 (+90, +393) | 31 | <0.0001 | +160 (+60, +320) | 36 | 0.0002 |
| BC = Black Carbon, CO = Carbon Monoxide, PM_2.5_ = Particulate Matter less than 2.5 microns in diameter, PNC = Particle Number Concentration, CI = Confidence Interval. | | | | | | |

#

# References

Chakrabarti B, Fine PM, Delfino R, Sioutas C. 2004. Performance evaluation of the active-flow personal dataram pm2.5 mass monitor (thermo anderson pdr-1200) designed for continuous personal exposure measurements. Atmospheric Environment 38:3329-3340.

Hagler GSW, Yelverton TLB, Vedantham R, Hansen ADa, Turner JR. 2011. Post-processing method to reduce noise while preserving high time resolution in aethalometer real-time black carbon data. Aerosol and Air Quality Research 11:539-546.

Kirchstetter TW, Novakov T. 2007. Controlled generation of black carbon particles from a diffusion flame and applications in evaluating black carbon measurement methods. Atmospheric Environment 41:1874-1888.

Langan Products Inc. 2006. Langan model t15n carbon monoxide measurer - sensor manual.
